# Household chaos and preschool migrant children’s self-regulation: the mediating role of parent–child conflict and the moderating role of mindful parenting

**DOI:** 10.3389/fpsyg.2025.1416040

**Published:** 2025-05-27

**Authors:** Huihui Zhu, Lina Shu, Xiaoying Wang, Zhechuan Xu

**Affiliations:** ^1^Faculty of Education, Northeast Normal University, Changchun, China; ^2^Educational Psychology Department, Texas A&M University, College Station, TX, United States; ^3^College of Child Development and Education, Zhejiang Normal University, Hangzhou, China

**Keywords:** preschool migrant children, household chaos, parent–child conflict, mindful parenting, self-regulation

## Abstract

Self-regulation is a foundational ability for children’s learning and socioemotional development. Household chaos, as an unavoidable physical environmental risk in the early growth environment of preschool migrant children, may significantly threaten the development of children’s self-regulation. Therefore, this study, based on the Family Stress Model and the Risk-Protective Factor Model, explores how household chaos affects the self-regulation of preschool migrant children through parent–child conflict and how mindful parenting moderates this relationship. Nine hundred and forty Chinese preschool migrant children and their families participated in this study. The results indicated that after controlling for factors including gender, age, and family socioeconomic status, household chaos was significantly negatively associated with children’s self-regulation ability. Parent–child conflict mediated the relationship between household chaos and the self-regulation of preschool migrant children. At the same time, mindful parenting modified the adverse effects of parent–child conflict on self-regulation, but as the level of parent–child conflict increased, its protective effect gradually decreased. These results suggest that household chaos and parent–child conflict are important risks for the early development of self-regulation in preschool migrant children. In addition, mindful parenting reduced the tension arising from parent–child conflict. These findings underscores the importance of addressing environmental stressors and promoting positive parent–child relationship in early childhood. Moreover, the results provide important implications for both practice and research.

## Introduction

Migrant children refer to children who accompany their parents from rural to urban areas for employment, and their household registration does not belong to the urban area (Ding et al., 2019). As China’s urbanization accelerates, the number of migrant workers moving from rural areas to cities continues to increase. According to the latest data from the seventh national population census by the National Bureau of Statistics in China (National Bureau of Statistics, 2021), the migrant population had significantly increased to 376 million. Among them, the number of migrant children in the 0–6 age group was approximately 23.51 million, accounting for 20.8% of the total population of children in the same age group in China. The data indicates that nearly one in every five preschool children is a preschool migrant child, who have become a group that cannot be ignored.

As a disadvantaged group in urban life, the cognitive and social development of migrant preschoolers has received extensive attention from researchers (Liang et al., 2021). Compared to urban preschoolers, migrant children demonstrated significantly higher rates of externalized problem behaviors (Li et al., 2015) and generally performed worse in areas like numeric operations, pre-literacy skills, and learning quality (Yan, 2017). These issues were likely tied to early self-regulation development, which was critical since difficulties in self-regulation were known to underlie common childhood learning and behavioral disorders, including dyslexia and ADHD. As the importance of self-regulation across various developmental stages gains recognition (Robson et al., 2020), it’s increasingly viewed as a crucial skill that supports positive early development. However, research on self-regulation has overlooked the migrant children population.

Previous research has found that children from disadvantaged backgrounds are often exposed to multiple risks within their family environments, which can threaten the early development of self-regulation (Evans and Kim, 2012). Among these risks, household chaos—a type of physical environmental risk within the family—encompasses adverse elements such as disorder, noise, and instability, and is likely to have a detrimental effect on children’s self-regulation abilities (Evans and Wachs, 2010). Migrant children’s parents often held temporary jobs and tended to rent homes in suburban areas or “urban villages,” where living spaces were typically characterized by narrowness, crowdedness, noise, and irregular family routines (Dan, 2016; Chang et al., 2016). These conditions were likely to expose migrant children to higher levels of household chaos. Therefore, this study aims to understand the relationship between the household chasos and self-regulation in preschool migrant children. The result will provide insights and recommendations for policymakers and families.

## Literature review and hypotheses

Self-regulation is the ability to effectively regulate cognition, emotions, and behavior in specific situations (Nigg, 2016). A preschooler with strong self-regulation abilities can control impulses, manage intense emotional reactions, maintain focus on tasks, and manipulate multiple pieces of information in memory. Previous research has found that self-regulation is highly sensitive to early adversity and risk (Blair and Raver, 2012). However, not all at-risk children show deficits in self-regulation. In fact, researchers are now calling for attention to the developmental differences in self-regulation and the necessity of understanding the interactions between different influencing factors, including risks and positive factors. For instance, the study conducted by Kochanska et al. (2009) demonstrated that secure attachment relationships can serve as a protective factor mitigating the adverse effects of genetic risk on young children’s self-regulation capabilities. Du Toit et al. (2020) also highlighted that children’s developmental trajectories were not uniform; rather, they were shaped by dynamic interactions between risk and protective factors. On one hand, risk factors such as household chaos and parent–child conflict may threaten the development of self-regulation in preschool migrant children. On the other hand, mindful parenting as an important protective factor may help buffer the adverse effects of risks on self-regulation. However, the interrelationships between these factors and their combined effects on the development of self-regulation in preschool children require further research.

### Household chaos and self-regulation

Household chaos describes as a home environment characterized by noise, crowding, and a lack of order and routine, reflecting the degree of disorganization within a family (Evans and Wachs, 2010). Early studies have shown that various indicators of household chaos are closely associated with self-regulation abilities. For instance, noise found to be associated with attention problems in preschool children, while a lack of routine was significantly linked to lower delay of gratification abilities (Martin et al., 2011). Recently, an increasing number of researchers have adopted the more convenient parent-reported Confusion, Hubbub, and Order Scale (CHAOS) to comprehensively assess multiple indicators of household chaos, including noise, irregularity, and disorganization (Zhang, 2022). Interestingly, findings from studies using the CHAOS tool have revealed inconsistent association between household chaos and children’s self-regulation abilities (Zhan and Guo, 2023; Andrews et al., 2021). Among these studies, household chaos was significantly associated with self-regulation levels only in preschool-aged children from low-income families. This suggests that household chaos may be an important risk factor for self-regulation in young children from disadvantaged backgrounds. Due to economic and life stressors, as well as the larger number of children in migrant families, children from these families often face more adverse factors in their home environments. The allostatic load model further posited that chronic stress could lead to dysregulation of the nervous system and abnormal reactivity (Brown et al., 2019), which may further resulted in poor self-regulation development (Blair and Raver, 2012). However, the association between household chaos and the self-regulation of migrant children remains underexplored in empirical research. Based on the above, this study puts forward the following hypothesis:

*Hypothesis 1*: Household chaos is negatively associated with the self-regulation of preschool migrant children.

### Mediating role of parent–child conflict

Parent–child conflict is a negative feature of parent–child relationships, encompasses arguments, disagreements, and physical conflicts between parents and children (Pianta et al., 1997). Previous research has found that various adverse environmental factors within families, such as poverty, parenting stress, maternal mental health issues, are closely associated with parent–child conflict (Ho et al., 2022; Garcia et al., 2017; Zou et al., 2019). Additionally, a longitudinal study further revealed that family financial strain and general stress have specific, negative, indirect effects on children’s self-regulation skills through increases in parent–child conflict (Duran et al., 2018). As the Family Stress Model (FSM) suggested, stressors in the family environment (such as economic stress and parenting stress) can be transmitted to children through negative interaction processes within the family, thereby affecting the children’s psychological well-being and behavioral performance (Masarik and Conger, 2017). Households with high levels of chaos are often characterized by excessive noise, crowded living spaces, and a lack of daily routines, which can be viewed as a stressful and adverse environment. The fatigue hypothesis also stated that family members who were chronically exposed to such negative environmental stimuli were more likely to experience increased fatigue, leading to more negative parent–child interactions (Wachs and Camli, 1991). Related studies have also shown that a noisy family environment is closely related to parent–child conflict (Coldwell et al., 2006). Parent–child conflict created emotional stress and undermined the secure relationships necessary for children to learn and practice self-regulation (Evans and Kim, 2012). Thus, parent–child conflict may serve as a potential mechanism through which household chaos is associated with the self-regulation of migrant preschool children. Based on this, the hypothesis is proposed in this study:

*Hypothesis 2*: Parent–child conflict mediates the relationship between household chaos and preschool migrant children’s self-regulation.

### Moderating role of mindful parenting

Mindful parenting is a positive parenting approach. It involves purposefully applying personal mindfulness to the parenting process, aiming to foster intentional, present-focused, and non-judgmental attention and awareness of both the child and one’s parenting behaviors (Duncan et al., 2009). Unlike risk factors such as household chaos and parent–child conflict, mindful parenting serves as a protective factor for young children’s self-regulation in the family environment. Existing research has demonstrated that mindful parenting is positively associated with children’s self-regulation abilities (Cheung et al., 2021). According to the risk-protective factor model, children’s developmental outcomes were shaped by the interplay between risk and protective factors, with protective factors serving to buffer the adverse effects of risks on child development (Fergus and Zimmerman, 2005). Consequently, mindful parenting may also mitigate the adverse effect of household chaos and parent–child conflict on the self-regulation of migrant children. Mindful parenting model suggested that mindful parenting helped parents pause during conflicts, regulate negative emotions, and avoid impulsive reactions when faced with children’s misbehavior (Gouveia et al., 2016). This process not only reduced escalations of conflict but also provided children with positive social modeling, thereby supporting their self-regulation development. Accordingly, this study proposes:

*Hypothesis 3*: Mindful parenting can buffer the adverse effects of parent–child conflict on the self-regulation of migrant preschool children.

Furthermore, mindful parenting can also help parents cope with environmental stress, particularly in high-pressure and challenging situations. Parents with higher levels of mindful parenting were more likely to remain calm and respond appropriately (Cheung and Wang, 2022). Previous studies have found that mindful parenting can serve as a protective factor for children’s developmental outcomes in challenging environments, such as mitigating the adverse effects of household chaos on children’s behavioral problems (Acet et al., 2024). Therefore, present study propose that certain migrant parents can sustain mindful parenting practices despite high levels of household chaos, thereby buffering the adverse effect of household chaos on children’s self-regulation. Accordingly, this study proposes:

*Hypothesis 4*: Mindful parenting can moderate the relationship between household chaos and the self-regulation of migrant preschool children.

Additionally, a substantial body of research has found that higher levels of mindful parenting were also strongly associated with higher-quality parent–child relationships (Coatsworth et al., 2018). In chaotic and crowded environments, parents with elevated levels of mindful parenting may still be able to engage in attentive listening, promptly recognize their children’s emotions and needs, and establish closer relationships with their children, thereby reducing the likelihood of parent–child conflict. Therefore, this study proposes:

*Hypothesis 5*: Mindful parenting may also buffer the adverse effect of household chaos on the level of parent–child conflict.

### The current study

This study focuses on preschool migrant children, aiming to delve into the family context to explore the mechanisms of household chaos, parent–child conflict, and mindful parenting on the self-regulation of these children.

To begin with, guided by the Family Stress Model, this study examines the relationship between household chaos and parent–child conflict as risk and stress factors, constructing a mediation model with parent–child conflict as a latent variable. This is because the Family Stress Model posited that within the family system, influencing factors can be categorized into proximal and distal stress factors. Generally, process factors within the family (such as negative parenting and parent–child conflict) are considered proximal stress factors, while non-process factors (such as family structure, socioeconomic status, and household chaos) are distal stress factors. Distal stress factors often exert their influence through proximal mechanisms. Therefore, compared to parent–child conflict, household chaos is more likely to be a distal stress factor, indirectly affecting the self-regulation of migrant children through the proximal mechanism of parent–child conflict.

**Figure 1:**
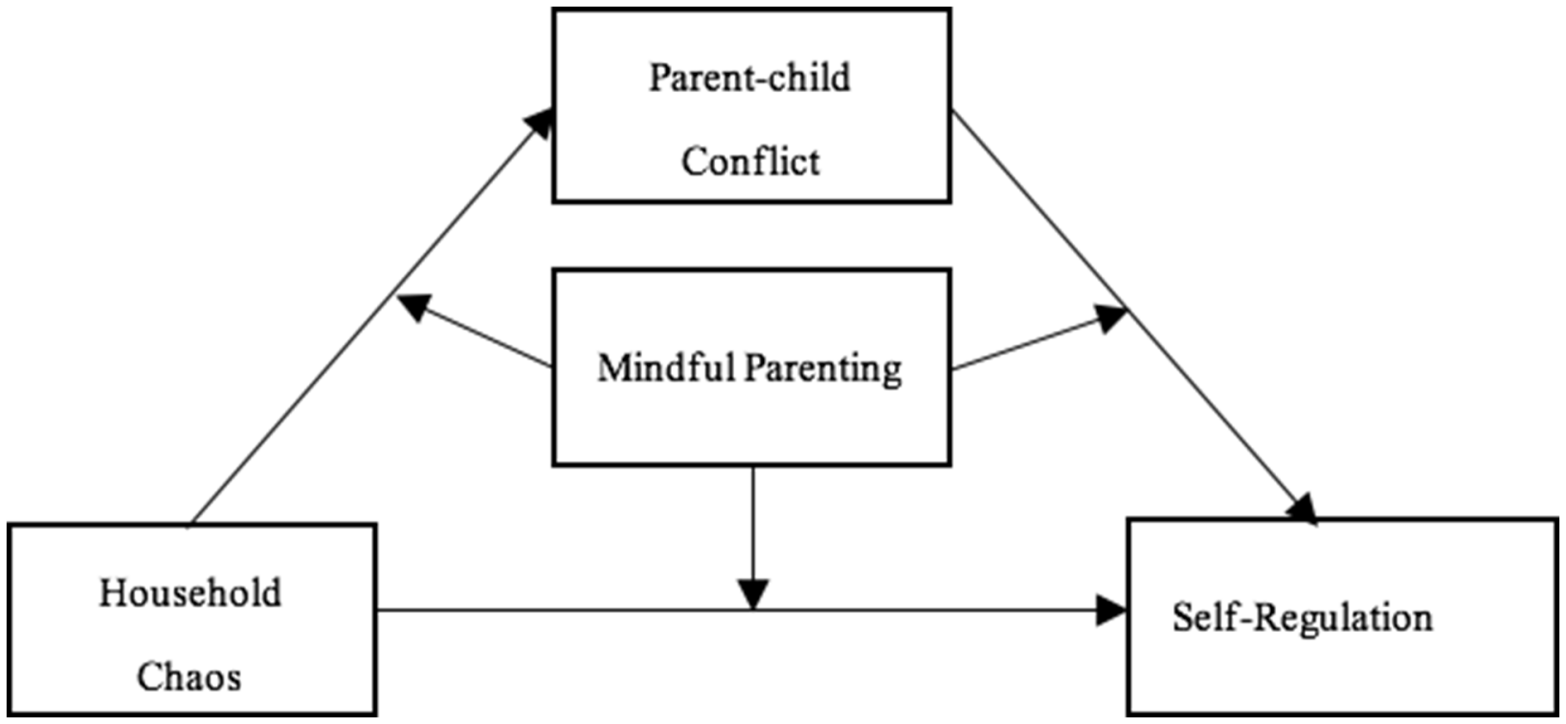
Hypothetical model.

Next, under the guidance of the Risk-Protective Factors Model, this study considers mindful parenting as a protective factor and household chaos and parent–child conflict as risk factors, exploring the interaction between mindful parenting and these two risk factors, and establishes a moderated mediation model (see Figure 1). Notably, the protective role of these factors can vary depending on the level of risk. On the one hand, the Stress-buffering Hypothesis (Landerman et al., 1989) posited that protective factors can effectively buffer the adverse effects of risk, regardless of the level of risk. On the other hand, the Stress-vulnerability Hypothesis suggested that the protective effect of these factors diminishes as the level of risk increases, meaning that under high-risk conditions, the protective effect gradually weakens (Vanderbilt-Adriance and Shaw, 2008). Hence, the specific protective mechanisms of mindful parenting in buffering the negative effects of household chaos and parent–child conflict on children’s self-regulation are a key focus of this study.

## Methods

### Participants

This study employed purposive sampling to select six kindergartens in Hangzhou, Jiaxing, and Dongyang cities in Zhejiang Province, where migrant preschool children are concentrated. Zhejiang Province has high concentration of migrant populations in China. In addition to Hangzhou, the provincial capital, Jiaxing and Dongyang are also economically developed cities where private preschools enroll a significant number of migrant children. Therefore, we selected these three cities and chose two private preschools in each, totaling six preschools for data collection. The decision to focus on these cities was driven by the research team’s goal to include as many cities and preschools as possible within our capacity to enhance the representativeness of the study. However, due to logistical constraints, the present study were only able to establish connections with private preschools in these three cities. The specific criteria for selecting the preschools were as: (1) The preschools had to enroll at least 100 migrant children, ensuring a sufficiently large sample size for our study; (2) The preschool administrators and parents had to agree to participate in and support the research.

A total of 1,287 parents of preschool children were surveyed using questionnaires. The recruitment process for participants was as follows: After identifying the six kindergartens, researchers first obtained consent and cooperation from the kindergarten principals and classroom teachers. Next, the purpose and objectives of the study were explained in detail to parents through WeChat groups for each class. Finally, parents who voluntarily agreed to participate in the study, after fully understanding its intentions, could access the online questionnaire via the Wenjuanxing platform on their mobile devices. All responses were collected anonymously. The questionnaire screening process involved three stages: firstly, based on the information provided by parents regarding the children’s household registration status, those not from migrant families were excluded, resulting in the removal of 273 preschool children. Secondly, surveys with excessively short completion times (less than 300 s) and exhibiting response patterns indicative of invalid responses (such as consistently selecting the same option) were deemed invalid, leading to the exclusion of 66 surveys. Thirdly, eight surveys were identified as completed by grandparents rather than parents and were consequently excluded. In total, 940 valid questionnaires were obtained from the original 1,257 surveys, yielding an effective response rate of 73.04%. The average age of the Children was 5.23 years (SD = 1.04), with 505 boys (53.7%) and 435 girls (46.3%). The average age of the parent participants was 34.54 years (SD = 4.71), with 189 father (20.1%) and 751mother (79.9%). Demographic information is provided in Table 1.

**Table 1:** Demographic information (*N* = 940).

Demographic variable	Group	Number	Percentage
Parent	Father	189	20.1%
	Mother	751	79.9%
Parental education	Primary school or below	32	3.4%
	Junior high school	401	42.7%
	High school	280	29.8%
	Bachelor’s degree	163	17.3%
	Postgraduate or above	64	6.8%
Parental occupation	Temporary workers, unemployed, or job-seeking individuals	248	26.4%
	Manual laborers (e.g., migrant workers)	374	39.8%
	General administrative staff and general professionals (e.g., salespersons)	234	24.9%
	Middle-level managers and mid-level professionals (e.g., doctors)	47	5.0%
	Senior managers and senior professionals (e.g., company managers)	37	3.9%
Family income	Blelow 3	79	8.4%
	30,001–50,000	144	15.3%
	50,001–100,000	329	35.0%
	100,001–150,000	176	18.7%
	150,001–200,000	126	13.4%
	20	86	9.1%
Child gender	Male	505	53.7%
	Female	435	46.3%
Child age	4 years and below	271	28.8%
	5 years	290	30.9%
	6 years and above	379	40.3%

### Measurements

#### Confusion, hubbub, and order scale

In this study, we utilized the Confusion, Hubbub, and Order Scale (CHAOS) developed by Matheny et al. (1995), to measure preschool migrant children’s household chaos. The scale has 15 items (e.g., There is often a fuss going on at our home). Respondents rated their experiences on a two-point Likert scale (1 = yes, 0 = no), measuring the degree to which daily home atmosphere is marked by a lack of routine, confusion, and noise. The total score indicates the level of home chaos, with higher scores denoting more disorganized, confused, and noisy home environments. The scale has demonstrated good reliability and validity in prior studies in China (Zhao and Liu, 2020). In our study, the Cronbach’s alpha for this scale was 0.73.

#### Parent–child conflict subscale

In order to measure the level of parent–child conflict, we employed the parent–child conflict subscale adapt by Zhang et al. (2008) from Child–Parent Relationship Scale (CPRS) (Pianta et al., 1997). This subscale consists of 11 items (e.g., My child easily gets angry with me) scored on a five-point scale (1 = Not applicable, 5 = Completely applicable). Higher scores indicate higher levels of parent–child conflict. This subscale has demonstrated strong reliability and validity in previous studies in China (Zhu et al., 2024). In this study, the Cronbach’s alpha for the parent–child conflict subscale was 0.85.

#### Interpersonal mindfulness in parenting scale

The assessment of mindful parenting in this study drew upon the Interpersonal Mindfulness in Parenting Scales (IM-P), originally developed by Duncan et al. (2009) subsequently revised by Pan et al. (2019). This instrument encompasses four dimensions: interacting with full attention, compassion and acceptance, self-regulation in parenting, and emotional awareness of child, constituting a total of 24 items (e.g., Stopping rather than reacting immediately when facing difficulties with child). Responses were rated on a five-point Likert scale (1 = Never, 5 = always). Higher scores on this scale represent heightened levels of parental mindfulness in the context of parenting. The scale has exhibited strong reliability and validity in previous studies in China (Wang et al., 2023). The internal consistency of this instrument, as assessed by Cronbach’s alpha, was 0.88 in the present study.

#### Child self-regulation and social behavior questionnaire

The questionnaire of child self-regulation utilized in this study is the Child Self-Regulation and Social Behavior Questionnaire (CSBQ), developed by Howard and Melhuish (2016). The questionnaire comprises seven sub-dimensions, namely cognitive self-regulation, emotional self-regulation, behavioral self-regulation, internalizing problems, externalizing problems, prosocial behavior, and social skills. In the current study, only the three self-regulation subscales of the CSBQ were used: cognitive self-regulation; emotional self-regulation; and behavioral self-regulation, encompassing a total of 17 items (e.g., emotionally stable and calm, easy to get along with). Responses were scored on a 5-point scale (1 = Not at all, 5 = Completely), with higher scores indicating stronger self-regulation abilities in children. The scale has demonstrated robust reliability and validity in prior research in China (Huang et al., 2022). The Cronbach’s alpha reliability coefficient for this scale in the present study was found to be 0.81. The Cronbach’s alpha coefficients for the cognitive, behavioral, and emotional self-regulation subscales were 0.77, 0.75, and 0.70, respectively.

#### Control variables—family socioeconomic status

Parental education level, parental occupation, and family annual income level were the three commonly indicators of family socioeconomic status (SES) (Larson et al., 2015). Among them, parental education level ranged from “primary school or below” to “master’s degree or above,” assigned scores from 1 to 6 respectively; parental occupation refers to the research by Shi and Shen (2007), ranging from “temporary workers, unemployed, or unemployed persons” to “senior management, senior professional and technical personnel, or managerial personnel,” assigned scores from 1 to 5 respectively; family annual income refers to the minimum wage standards in Zhejiang Province in 2023, ranging from “below 30,000 yuan” to “over 200,000 yuan,” assigned scores from 1 to 6, respectively. The Programme for International Student Assessment (PISA) (2002) was adopted for SES calculation: first, standardized the three indicators, then conducted principal component analysis, and finally, according to the formula SES = (β1 × Z parental occupation + β2 × Z parental education level + β3 × Z family annual income level) / f, the specific value of SES is calculated, where β1, β2, β3 are factor loadings, and f is the characteristic root of the first factor. Lastly, the calculation formula of family SES status in this study is: SES = (0.75 × Z parental occupation + 0.78 × Z parental education level + 0.76 × Z family annual income level) / 0.58.

### Data analysis

Since the data were solely collected from parental reports, there may be issues related to common method bias. To address this, the study conducted Harman’s single-factor test in SPSS 22.0 for all variables involved. The results indicated that 14 factors had eigenvalues greater than 1, with the first factor explaining 15.55% of the variance, which was below the 40% threshold. Therefore, the study was free from significant common method bias.

Reliability analysis, descriptive statistics, and correlation analysis of the questionnaires were conducted using SPSS 22.0. First, since all questionnaires used Likert scales, after reverse-coding the negatively worded items, the reliability levels of each variable were calculated. Then, descriptive statistics and correlation analyses were performed by computing the means of variables such as household chaos, parent–child conflict, mindful parenting, and self-regulation.

The structural equation modeling analysis was conducted in Mplus 8.0. Before analysis, gender (boys = 1, girls = 0) was dummy-coded, and other continuous variables (e.g., the independent variable household chaos, the dependent variable self-regulation, the mediator parent–child conflict, and the moderator mindful parenting) were standardized. Firstly, the researcher included the control variables (gender, age and SES), the independent variable—household chaos, and the dependent variable —self-regulation in the structural equation model (SEM) to test the main effect of household chaos. Nextly, the researcher further incorporated the mediator—parent–child conflict in the model to conduct the mediated model analysis. Finally, the moderator—mindful parenting was added to the model to test the moderated mediation effect, examining the moderating role of mindful parenting across the mediation paths. Parameter estimation was conducted using the bootstrap method with 1,000 samples. A significant effect was confirmed if the 95% confidence interval [BootLLCI, BootULCI] did not include zero.

To assess the goodness of the fit of the model, the following fit indices were chosen, such as Chi-square/df Ratio, Comparative Fit Index (CFI), Tucker-Lewis index (TLI), Standardized Root Mean Square Residual (SRMR), and Root Mean Square Error of Approximation (RMSEA). The cutoff values of χ^2^/df ≤ 5, CFI ≥ 0.95, TLI ≥ 0.90, SRMR ≤ 0.08 and RMSEA ≤ 0.06 were adopted as the good fit criteria in this study (Schreiber et al., 2006). When individual data points did not meet the criteria, the model fit was still acceptable.

## Results

### Descriptive statistics and correlation analysis

Household chaos was significantly positively correlated with parent–child conflict and significantly negatively correlated with preschool migrant children’s self-regulation and mindful parenting. Parent–child conflict showed significant negative correlations with both preschool migrant children’s self-regulation and mindful parenting. Additionally, gender, age, and family SES were significantly correlated with household chaos, parent–child conflict, and self-regulation. As a result, these three factors (gender, age, and family SES) were included as control variables in subsequent analyses. For detailed results, see Table 2.

**Table 2:** Descriptive statistics and correlation analysis (*N* = 940).

Variables	1	2	3	4	5	6	7	8	9	10
1. Gender	–									
2. Age	0.05	–								
3. SES	−0.02	−0.14^**^	–							
4. Household chaos	−0.02	0.01	−0.09^**^	–						
5. Parent–child conflict	0.02	0.00	−0.11^***^	0.44^***^	–					
6. Emotional self-regulation	0.01	0.05	0.07^*^	−0.26^***^	−0.46^***^	–				
7. Behavioral self-regulation	−0.09^**^	0.13^***^	0.08^*^	−0.31^***^	−0.44^***^	0.51^***^	–			
8. Cognitional self-regulation	−0.05	0.12^***^	0.05	−0.28^***^	−0.33^***^	0.36^***^	0.53^***^	–		
9. Self-regulation	−0.06	0.13^***^	0.09^*^	−0.36^***^	−0.51^***^	0.77^***^	0.85^***^	0.78^***^	–	
10. Mindful parenting	−0.01	−0.03	0.19^***^	−0.33^***^	−0.41^***^	0.25^***^	0.30^***^	0.31^***^	0.36^***^	–
*M*	0.54	5.23	0.00	3.42	25.79	19.69	22.07	17.49	59.25	87.60
SD	0.50	1.04	3.02	2.79	8.48	3.55	3.73	3.62	8.75	12.32

*p < 0.05, **p < 0.01, ***p < 0.001.

### Direct effect of household chaos on self-regulation

To examine the main effect of household chaos on migrant preschool children’s self-regulation, a structural equation model (SEM) was constructed. The model demonstrated good fit: χ^2^/df = 2.05, CFI = 0.99, TLI = 0.98, RMSEA = 0.033, SRMR = 0.021. After controlling for gender, age, and SES, household chaos was significantly negatively correlated with self-regulation (β = −0.39, *p* < 0.001), supporting Hypothesis 1.

### Mediating role of parent–child conflict

**Figure 2:**
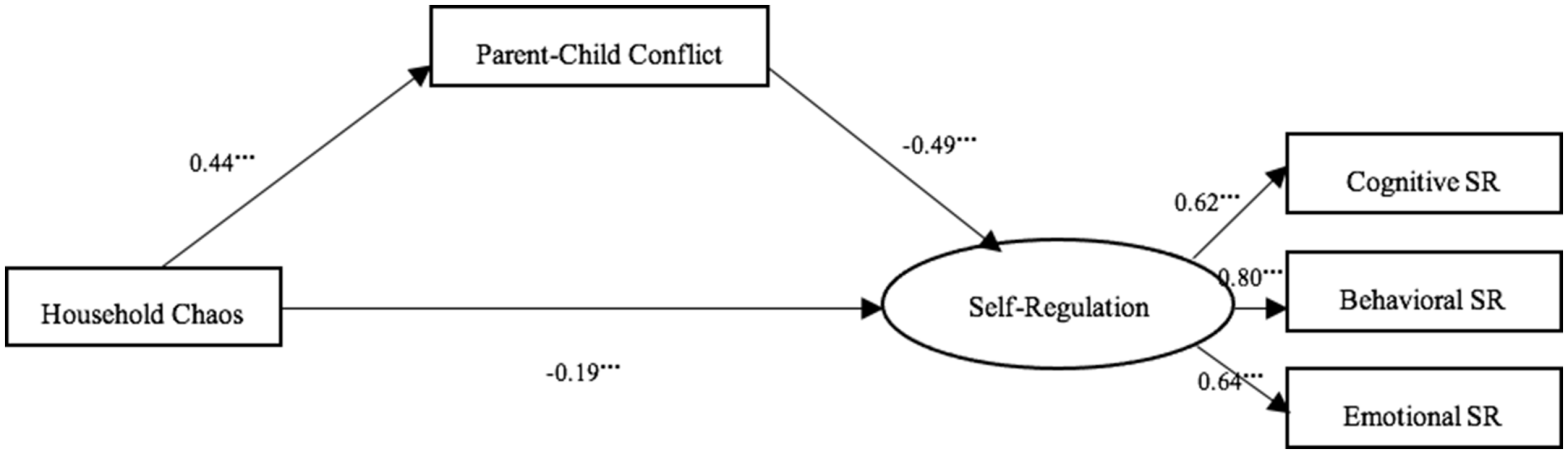
The mediating effect of parent–child conflict between household chaos and self-regulation. ***p < 0.001, **p < 0.01, *p < 0.05. Gender, age, and SES were control variables, which are not shown in figure, for concise purpose.

Next, parent–child conflict was included as a mediator, and the SEM (Figure 2) showed good fit: χ^2^/df = 4.28, CFI = 0.96, TLI = 0.94, RMSEA = 0.059, SRMR = 0.032. After controlling for covariates, household chaos was significantly negatively associated with self-regulation (β = −0.19, *p* < 0.001) and positively associated with parent–child conflict (β = 0.44, *p* < 0.001). Parent–child conflict, in turn, was significantly negatively related to self-regulation (β = −0.49, *p* < 0.001). As shown in Table 3, bootstrap analysis (95% CI [−0.26, −0.18]) confirmed the mediating role of parent–child conflict, with an indirect effect of 0.22, accounting for 54% of the total effect (0.41). Thus, Hypothesis 2 was supported.

**Table 3:** Mediating effect of parent–child conflict.

Pathway	Indirect effect	Boot SE	Boot LLCI Lower bound	Boot ULCI Higher bound	Relative mediation effect
Household chaos—parent–child conflict—self-regulation	−0.22	0.021	−0.26	−0.18	54%

### Moderating role of mindful parenting

**Figure 3:**
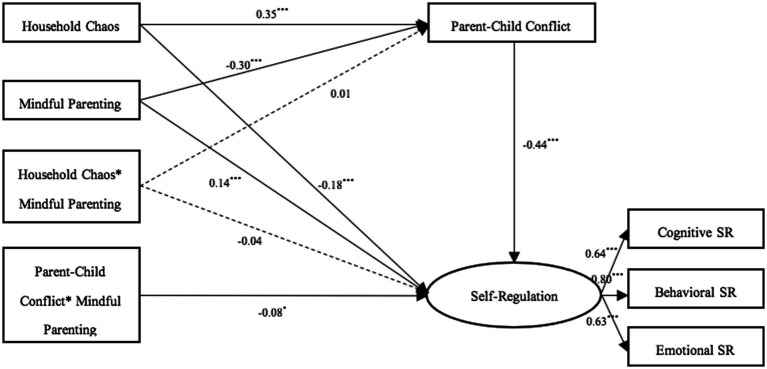
The moderating role of mindful parenting. All coefficients are standardized. Gender, age, and SES were controlled but omitted for simplicity. Dashed paths (---) in figures indicate nonsignificant effects. ***p < 0.001, **p < 0.01, *p < 0.05.

Finally, mindful parenting and its interaction terms with household chaos and parent–child conflict were added to the SEM (Figure 3). The model fit was acceptable: χ^2^/df = 4.13, CFI = 0.95, TLI = 0.92, RMSEA = 0.058, SRMR = 0.032. Results showed that the interaction between household chaos and mindful parenting was not significantly associated with parent–child conflict (β = 0.01, *p* > 0.05) or self-regulation (β = −0.04, *p* > 0.05). However, the interaction between parent–child conflict and mindful parenting was significantly associated with self-regulation (β = −0.08, *p* < 0.05), supporting Hypothesis 3.

**Figure 4:**
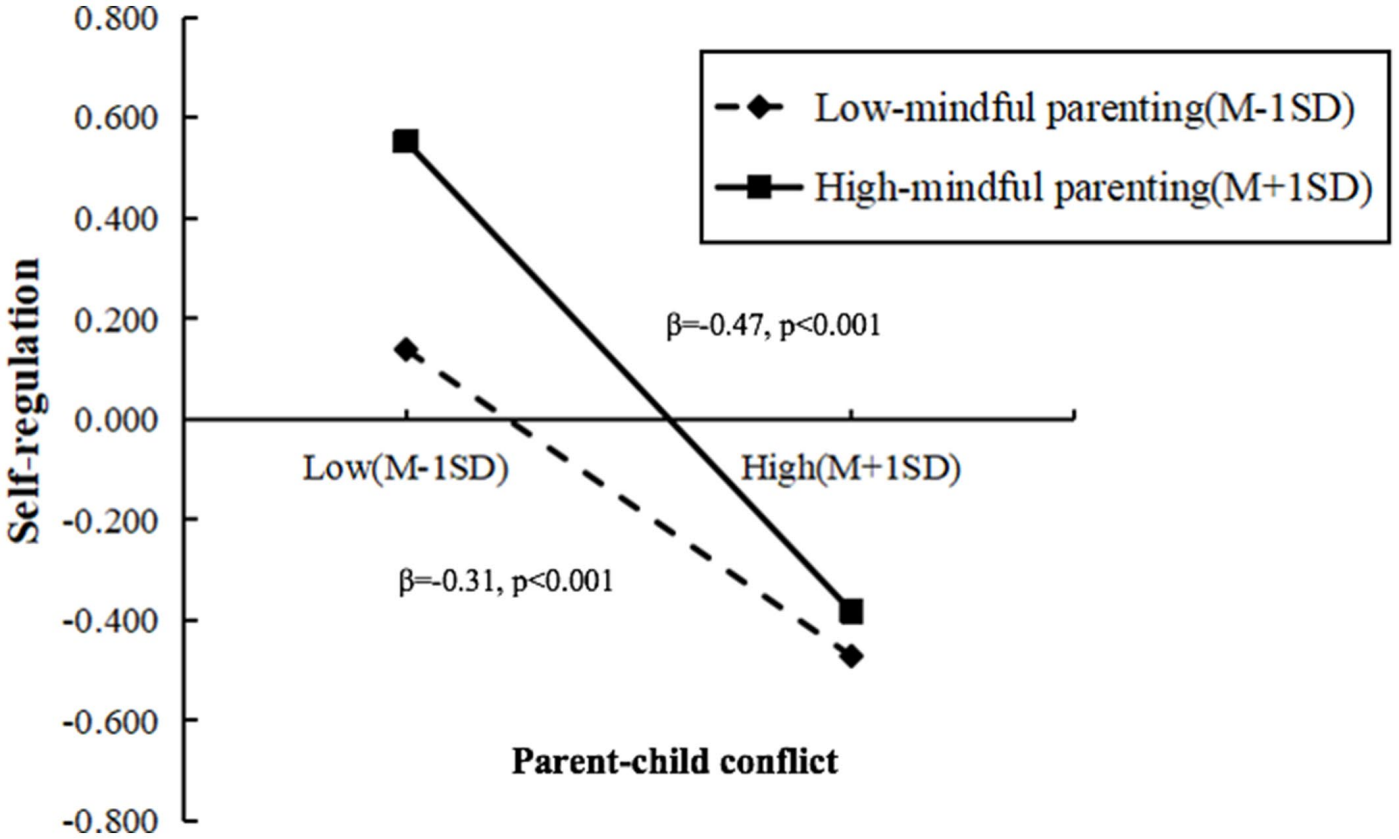
Relationship between parent–child conflict and self-regulation at different level of mindful parenting.

Additionally, we conducted a simple slope analysis to further elucidate the interaction effect. The results revealed that mindful parenting moderates the relationship between parent–child conflict and self-regulation in preschool migrant children. Specifically, in the low-mindful parenting group, increased parent–child conflict was significantly associated with decreased self-regulation (β = −0.31, *p* < 0.001), while in the high-mindful parenting group, the negative effect of parent–child conflict on self-regulation intensified (β = −0.47, *p* < 0.001). As depicted in Figure 4, the self-regulation abilities in the high-mindful parenting group were significantly superior to those in the low-mindful parenting group. However, with an increase in parent–child conflict, this advantage gradually diminished. This suggests as the level of parent–child conflict increases, the protective effect of mindful parenting on self-regulation diminishes.

## Discussion

### The association between household chaos and preschool migrant children

The current study revealed that household chaos was negatively associated with the self-regulation abilities of preschool migrant children. In other words, as the degree of household chaos increases, the self-regulation abilities of preschool migrant children tend to decline. This finding is consistent with earlier studies on low-income families, which have shown that household chaos is strongly linked to children’s ability to manage their thoughts and emotions (Berry et al., 2016; Zhan and Guo, 2023). This is likely because families with fewer resources often experience more chaotic home environments, filled with disorder, noise, and unpredictability, which may.

increase negative stimuli in the environment in which children grow up (Marsh et al., 2020). Meanwhile, for young children, who have not yet fully developed the attentional and regulatory capacities for discriminating irrelevant stimuli, they can be easily distracted by irresistible, changing external stimuli (Vernon-Feagans et al., 2016), thus increasing the burden of regulation in aspects such as attention, emotion, and behavior. Migrant families, as a socioeconomically disadvantaged group, frequently encounter systemic resource limitations—including financial precarity, constrained access to support services, and residential instability. These challenges, which parallel those observed in low-income households, often result in less structured and more chaotic home environments. Critically, such conditions may elevate developmental risks for preschool-aged children, particularly in the domain of self-regulation.

Therefore, the current study further confirms that even after controlling for other family variables (such as socioeconomic status), household chaos remains a significant and unique risk factor in low-income families, affecting the early development of self-regulation in preschool migrant children. These findings suggest multi-level interventions: at the family level, parents should select housing conducive to child development while actively reducing noise pollution through avoiding loud disputes, regulating electronic volumes, and establishing quiet zones; community organizations can support these efforts by distributing noise-reducing materials (e.g., rugs, sound panels) and offering mobile coaching on home organization; meanwhile, local governments should implement policy measures including designating enforced quiet zones and subsidizing soundproofing upgrades in family housing - collectively creating calmer, more structured environments to support migrant children’s self-regulation development.

### The mediating effect of parent–child conflict

The current study demonstrated that parent–child conflict played a mediating role in the relationship between household chaos level and self-regulation in preschool migrant children. This result is consistent with previous research, which has shown that household chaos is not only directly associated with self-regulation but can also indirectly affect children’s self-regulation through family interaction processes (Andrews et al., 2021). This could be attributed to the fact that in a noisy environment, parents in migrant families may act in ways that are harmful to child development, such as exhibiting low reactivity, limited communication, providing less support, and increased interference (Wachs, 1993). These behaviors, in turn, can increase the frequency of parent–child conflicts (Barnes et al., 2013), and children who in conflict situations may struggle to concentrate, inhibit their own actions, thus demonstrate poorer regulatory abilities (Vernon-Feagans et al., 2016).

These findings further corroborate the fundamental proposition of the Family Stress Model (Pearlin, 1989): chaotic and disorganized home environments, as a distal stressor, can negatively influence children’s self-regulation through mediating processes of adverse family interactions. Specifically, parent–child conflict as a proximal stressor, serving as a key mechanism through which distal environmental stressors affect young children’s self-regulatory capacities. This underscores the need for parents and educators to recognize the detrimental impact of negative parent–child interactions on early self-regulation development.

For migrant families, migrant parents should actively cultivate a harmonious and structured family environment. Particularly in stressful situations (e.g., noisy surroundings), they can employ stress-reduction techniques such as taking brief breaks or communicating with their partner to decompress. These practices help prevent stress transmission to children and reduce parent–child conflicts. At the institutional level, governments and kindergartens should provide parents with targeted support in stress management and parent–child communication. For instance, community-based workshops featuring role-playing activities could help migrant parents acquire nonviolent communication strategies, learn to respond patiently to children’s needs, and ultimately improve the quality of parent–child interactions.

### The moderating effect of mindful parenting

First, this study found that mindful parenting does not buffer the adverse effects of household chaos on parent–child conflict or self-regulation. Instead, it moderated the relationship between parent–child conflict and the self-regulation of migrant preschool children. This suggests that mindful parenting, as a supportive parenting resource, is more effective in mitigating the risks associated with parent–child conflict but has limited effectiveness in buffering the adverse effect of household chaos. This may be because the core of mindful parenting lies in enhancing parents’ emotional awareness and self-regulation abilities, and effectively improving parent–child relationships (Duncan et al., 2009). This can significantly reduce parent–child conflict in migrant families and, in turn, protect the self-regulation abilities of preschool children. In contrast, household chaos, as a physical environmental risk at the family level, exerts its negative effects on parent–child conflict and self-regulation primarily through direct exposure to adverse environmental stimuli (Evans and Wachs, 2010). These direct negative effects are more difficult to mitigate through mindful parenting.

Moreover, it is noteworthy that as the level of parent–child conflict escalates, the protective effect of mindful parenting gradually diminishes rather than strengthens. This pattern suggests that the buffering role of mindful parenting aligns more closely with the Stress-Vulnerability Hypothesis (Vanderbilt-Adriance and Shaw, 2008), which posits that protective factors tend to lose their effectiveness under conditions of high risk. In other words, intense parent–child conflicts substantially undermine the protective capacity of mindful parenting practices.

The findings suggest that for migrant parents, only improving mindful parenting may not be enough to handle intense parent–child conflicts. Parents also need to actively manage conflicts in daily parenting to prevent them from escalating into frequent, high-intensity confrontations. Specifically, migrant parents can actively participate in community-organized parenting workshops or support groups, and systematically learn mindful parenting skills and conflict resolution strategies. For example, by practicing mindfulness techniques, such as, pause-breathe-respond, parents can better regulate their emotions during conflicts. This helps reduce intense confrontations and maintain healthy parent–child relationships. Meanwhile, kindergartens and communities should provide professional support for high-conflict families. This includes counseling services or family interventions to help parents apply mindfulness techniques in real-life parenting situations. Therefore, for migrant families, only by combining mindful parenting with conflict management can they form a comprehensive intervention strategy to more effectively support children’s self-regulation development.

## Limitation and future direction

This study has several limitations. First, the cross-sectional nature of the data prevents us from establishing causal relationships, meaning our findings can only describe associations between variables rather than causality. Although we cannot directly infer causality, our goal is to explore potential associations between variables and lay the groundwork for future longitudinal studies. Future research could build on this study by examining the longitudinal relationships between household chaos, parent–child conflict, and the self-regulation of migrant preschool children within the sociocultural context of China.

Second, the data in this study were sourced solely from parental reports. Although common method bias tests did not indicate significant issues with the survey method, the information provided by parents may still deviate from reality. Parents might underestimate levels of household chaos or parent–child conflict, potentially leading to less objective results. Therefore, future studies should consider incorporating multiple assessment methods, such as observational measures and reports from children, teachers, and peers, to obtain more objective and comprehensive measurements.

Thirdly, the data collection in this study focused solely on family-level information and did not include data on kindergarten or classroom environments, nor did it employ nested model designs. As a result, the findings may not fully capture the potential influence of kindergarten and classroom settings on children’s self-regulation abilities, limiting the interpretability and applicability of the results. Future research should consider adopting multi-level or nested models to more comprehensively assess the combined effects of family, kindergarten, and classroom environments on child development.

Lastly, the sampling was limited to six kindergartens in three cities in Zhejiang Province and did not achieve nationwide coverage. This limited scope made it difficult to comprehensively reflect the actual situations of migrant families in different regions. Future research could consider expanding the sample size to include more areas and different types of kindergartens, thereby enhancing the generalizability and reliability of the findings.

## Conclusion

Our study is based on the Family Pressure Model and the Risk-protective Factor Model and research on preschool migrant children in Zhejiang Province, China. We conclude: First, after controlling for age, gender, and family SES status, household chaos was significantly and negatively associated with the self-regulation of preschool migrant children; Second, parent–child conflict mediated the relationship between household chaos and the self-regulation of preschool migrant children; Finally, parental mindful parenting moderated the relationship between parent–child conflict and the self-regulation of preschool migrant children.

## Data Availability

The original contributions presented in the study are included in the article/supplementary material, further inquiries can be directed to the corresponding author.
